# Heparanase—A Link between Coagulation, Angiogenesis, and Cancer

**DOI:** 10.5041/RMMJ.10069

**Published:** 2012-01-31

**Authors:** Yona Nadir, Benjamin Brenner

**Affiliations:** Thrombosis and Hemostasis Unit, Department of Hematology, Rambam Health Care Campus and Technion–Israel Institute of Technology, Haifa, Israel

**Keywords:** Heparanase, tissue factor, tissue factor pathway inhibitor, cancer

## Abstract

Heparanase that was cloned from and is abundant in the placenta is implicated in cell invasion, tumor metastasis, and angiogenesis. Recently we have demonstrated that heparanase may also affect the hemostatic system in a non-enzymatic manner. Heparanase was shown to up-regulate tissue factor (TF) expression and interact with tissue factor pathway inhibitor (TFPI) on the cell surface, leading to dissociation of TFPI from the cell membrane of endothelial and tumor cells, resulting in increased cell surface coagulation activity. More recently, we have shown that heparanase directly enhances TF activity, resulting in increased factor Xa production and activation of the coagulation system. Data indicate increased levels and possible involvement of heparanase in vascular complications in pregnancy. Taking into account the prometastatic and proangiogenic functions of heparanase, overexpression in human malignancies, and abundance in platelets and placenta, its involvement in the coagulation machinery is an intriguing novel arena for further research.

## INTRODUCTION

Cancer patients have a prothrombotic state because of the ability of cancer cells to activate the coagulation system and to interact with hematopoietic cells, thus tilting the balance between procoagulants and anticoagulants.^1^ Overexpression of tissue factor (TF), cancer procoagulant—a cysteine protease that activates factor X—and acquired activated protein C resistance,^2^ are thought to be the main factors for coagulopathy in malignant disorders. TF is a transmembrane receptor that is constitutively expressed in tumors, i.e. human leukemias, lymphomas, adenocarcinomas, and sarcomas.^3^ TF also plays a role in cellular signaling, contributing to tumor growth and metastasis.^3^,^4^ The only known endogenous modulator of blood coagulation initiated by TF is tissue factor pathway inhibitor (TFPI)—a plasma Kunitz-type serine protease inhibitor.^5^,^6^ Growing evidence suggests the involvement of tumor-derived proteins, including heparanase, in activation of the coagulation system.

## HEPARANASE

Heparanase is an endo-β-d-glucuronidase capable of cleaving heparan sulfate (HS) side chains at a limited number of sites, yielding HS fragments of still appreciable size (∼5–7 kDa).^7^,^8^ Heparanase activity has long been detected in a number of cell types and tissues. Importantly, heparanase activity correlated with the metastatic potential of tumor-derived cells, attributed to enhanced cell dissemination as a consequence of HS cleavage and remodeling of the extracellular matrix (ECM) barrier.^9^,^10^ Similarly, heparanase activity was implicated in neovascularization, inflammation, and autoimmunity, involving migration of vascular endothelial cells and activated cells of the immune system.^9^–^11^ A single human heparanase cDNA sequence was independently reported by several groups.^12^–^15^ Thus, unlike the large number of proteases that can degrade polypeptides in the ECM, one major heparanase appears to be used by cells to degrade the HS side chains of HS proteoglycans. Expression of heparanase is restricted primarily to the placenta, keratinocytes, platelets, and activated cells of the immune system, with little or no expression in connective tissue cells and most normal epithelia.^9^,^10^ Up-regulated expression of heparanase was noted in essentially all human tumors examined, as well as in inflammation, wound healing, and diabetic nephropathy.^9^–^11^ During embryogenesis, the enzyme is preferentially expressed in cells of the developing vascular and nervous systems.^16^

## PROMETASTATIC PROPERTIES OF HEPARANASE

The clinical significance of the enzyme in tumor progression emerges from a systematic evaluation of heparanase expression in primary human tumors. Immunohistochemistry, *in-situ* hybridization, RT-PCR, and real-time PCR analyses revealed that heparanase is up-regulated in essentially all human tumors examined. These include carcinomas of the colon,^17^,^18^ thyroid,^19^ liver,^20^ pancreas,^21^,^22^ bladder,^23^,^24^ cervix,^25^ breast,^26^ gastric,^27^,^28^ prostate,^29^ head and neck,^30^,^31^ as well as multiple myeloma,^32^ leukemia, and lymphoma.^33^ In most cases, elevated levels of heparanase were detected in about 50% of the tumor specimens, with a higher incidence in pancreatic (78%) and gastric (80%) carcinomas, and in multiple myeloma (86%). In all cases, normal tissue adjacent to the malignant lesion expressed little or no detectable levels of heparanase, suggesting that epithelial cells do not normally express the enzyme. In several carcinomas, most intense heparanase staining was localized to the invasive front of the tumor,^23^,^28^,^30^ supporting a role for heparanase in cell invasion. Furthermore, patients that were diagnosed as heparanase-positive exhibited a significantly higher rate of local and distant metastasis as well as reduced postoperative survival, compared with patients that were diagnosed as heparanase-negative.^18^,^22^,^23^,^28^,^32^ Collectively, these studies provide strong clinical support for the prometastatic function of heparanase. Interestingly, patient survival was noted to correlate not only with heparanase levels, but also with its localization. In addition to its presence in the cytoplasm, heparanase was also noted to assume nuclear localization, demonstrated by cell fractionation,^34^ and by immunostaining of cultured cells^34^ and tumor biopsies.^27^,^35^ Interestingly, nuclear localization was correlated with maintained cellular differentiation^35^ and favorable outcome of patients with gastric^27^,^35^ and head and neck carcinomas,^36^ suggesting that heparanase is intimately involved in gene regulation. Whether gene transcription and maintained cellular differentiation is due to direct interaction of heparanase with the DNA or is a consequence of heparanase-mediated nuclear-HS degradation is yet to be demonstrated. In addition, heparanase up-regulation in primary human tumors correlated in some cases with larger tumors,^20^,^26^,^28^ and with enhanced microvessel density,^18^,^20^,^24^,^32^ providing clinical support for the proangiogenic function of the enzyme.

## HEPARANASE POLYMORPHISMS

Heparanase gene single nucleotide polymorphisms (SNPs) were characterized in Jewish populations of Israel.^37^ Four Israeli Jewish populations (Ashkenazi, North African, Mediterranean, and Near Eastern) were examined for seven heparanase gene SNPs. Four out of seven SNPs were found to be polymorphic. Population comparisons revealed significant differences in SNPs allele frequency between Near Eastern and each of the other three populations. Genotype and allele frequencies in Jewish populations were different from non-Jewish populations, except for a certain similarity to Caucasians.^37^ Ostrovsky et al. found an association of heparanase gene SNPs with hematological malignancies.^38^ Genotype frequency comparisons revealed a significant association of specific SNPs with multiple myeloma (MM), acute myeloid leukemia (AML), and acute lymphoblastic leukemia (ALL) patients. Examination of heparanase gene mRNA expression by real-time RT-PCR indicated a significantly lower heparanase expression level in ALL patients and a higher expression level in MM and AML patients, compared to healthy controls.^38^ The findings were not verified in ALL patients from Northern Ireland.^39^ Ralph et al. reported on an association between a specific heparanase SNP and stage of ovarian cancer disease, while the association was not found in vascular endothelial growth factor (VEGF) SNPs.^40^ Further research is needed to explore the clinical relevance of heparanase polymorphism detection.

## INTERACTION OF HEPARANASE WITH HEPARINS

Anticoagulant activities of cell surfaces have been predominantly attributed to HS,^41^,^42^ which is composed of repeating hexuronic and D-glucosamine sulfated disaccharide units. HS has been shown to exert anticoagulant activities on cells, on ECM, and in tissues due to its catalyzing function for protease inhibition by antithrombin and subsequent complex formation.^41^–^43^ Moreover, cell surface HS can facilitate the catabolism of coagulation factors such as factor VIII.^44^ Other coagulation inhibitors such as TFPI also associate with the luminal face of the endothelial cell plasma membrane via HS.^45^ HS is also important constituents of the subendothelial basement membrane, where they cross-link various components, e.g. laminin and collagens, thereby contributing to the integrity of the blood vessel wall.^46^ HS, unfractionated heparin, and other heparin derivatives have been investigated as heparanase inhibitors, and some of them exerted antimetastatic activity in animal models.^47^ Both the type of the polysaccharide backbone and the degree of sulfation seem to affect the heparanase-inhibiting activity of sulfated polysaccharides.^48^,^49^ However, different heparin preparations display significantly different antiheparanase activity,^48^,^49^ indicating that this activity is also dependent on more subtle structural features. Recently, heparanase’s strong affinity to heparins was utilized *in vitro* to reverse heparin’s effect. Heparanase was shown to reverse the anticoagulant activity of unfractionated heparin on the coagulation pathway as well as on thrombin activity. In addition, heparanase abrogated the factor Xa inhibitory activity of low-molecular-weight heparin (LMWH). The procoagulant effects of heparanase were also exerted by its major functional heparin-binding peptide.^50^

## HEPARANASE AS A CO-FACTOR TO TF ACTIVITY

Tissue factor is constitutively expressed in various cell types, including pericytes adjacent to the vessel wall, but absent from the blood cell and endothelial cell surface. This localization is crucial for hemostasis since it prevents a direct contact between TF and the circulating blood. Immunohistochemical studies revealed that many tumors express high levels of TF, including leukemia cells,^51^ raising the possibility of a TF role in the pathogenesis of cancer.^1^ We have demonstrated that heparanase overexpression in human leukemia, glioma, and breast carcinoma cells results in a marked increase in TF levels verified by immunoblot and real-time PCR analyses.^52^ Likewise, TF was induced by exogenous addition of recombinant heparanase to tumor cells and primary endothelial cells, induction that was mediated by p38 phosphorylation and correlated with enhanced procoagulant activity. TF induction was further confirmed in heparanase-overexpressing transgenic mice and, moreover, correlated with heparanase expression levels in leukemia patients.^52^ Lately, heparanase was found to exert also non-enzymatic activities, independent of its involvement in ECM degradation and alterations in the extracellular microenvironment.^53^ For example, inactive heparanase enhances Akt signaling and stimulates PI3K- and p38-dependent endothelial cell migration and invasion.^54^ It also promotes VEGF expression via the Src pathway.^55^ Up-regulation of TF adds another example of the multiple non-enzymatic functions of heparanase. Recently, we have demonstrated that heparanase may serve as a co-factor of TF, suggesting that heparanase is directly involved in activation of the coagulation cascade.^56^ The findings were supported by experiments indicating that heparanase increases the level of factor Xa in the presence of TF/VIIa and the effect is enzymatically independent. The newly generated Xa had the same molecular weight as Xa cleaved by TF/VIIa and was active as depicted by increased conversion of prothrombin to thrombin. Increased Xa generation in the presence of heparanase was shown to be relevant in the clinical setting. Thus, apart from the ability of heparanase to increase Xa levels in normal human plasma, a statistically significant positive correlation was found in patients with acute leukemia and healthy donors between the plasma levels of heparanase and Xa.^56^

## HEPARANASE AND TFPI

TFPI is a plasma Kunitz-type serine protease inhibitor and the only known endogenous modulator of blood coagulation initiated by TF.^5^,^6^ TFPI concentration in plasma is increased in patients with acute myocardial infarction.^57^,^58^ There are also reports on the plasma levels of TFPI in relation to disseminated intravascular coagulation^59^ and to other diseases, such as diabetes mellitus,^60^ renal diseases,^61^ and cancer.^62^,^63^ Recently we demonstrated that exogenous addition or overexpression of heparanase by transfected cells resulted in release of TFPI from the cell surface and its accumulation in the cell culture medium.^64^ Importantly, the *in-vitro* studies were supported by elevation of TFPI levels in the plasma of transgenic mice overexpressing heparanase. Moreover, increased levels of TFPI have been noted in the plasma of cancer patients,^62^,^63^ reflecting, possibly, induction of heparanase expression and elevation of its plasma levels revealed by a newly developed ELISA assay.^65^ In human umbilical vein endothelial cell (HUVEC) and tumor-derived cell lines, release of TFPI from the cell surface correlated with enhanced TF-mediated coagulation. This effect was evident already 30 min following heparanase addition and prior to the induction of TF^52^ or TFPI expression. Thus, heparanase enhances local coagulation activity by two independent mechanisms: induction of TF expression^52^ and TFPI dissociation from the cell surface. Both functions require secretion of heparanase, but not its enzymatic activity. The underlying mechanism is apparently release of TFPI due to its physical interaction with the secreted heparanase, as clearly evident by co-immunoprecipitation experiments,^64^ reflecting a functional interaction between heparanase and a membrane protein.

Elevated levels of heparanase may be generated locally upon degranulation of neutrophils, mast cells, and platelets,^66^ further facilitating blood coagulation at the site of platelet activation. The hemostatic function of heparanase, executed by inducing TF expression and releasing TFPI from the endothelial cell surface, provides a mechanism by which heparanase contributes to tumor complication, in addition to its established proangiogenic and prometastatic activities.^67^,^68^

## A MODEL FOR INTERACTION BETWEEN HEPARANASE, TF, AND TFPI

**Figure 1:**
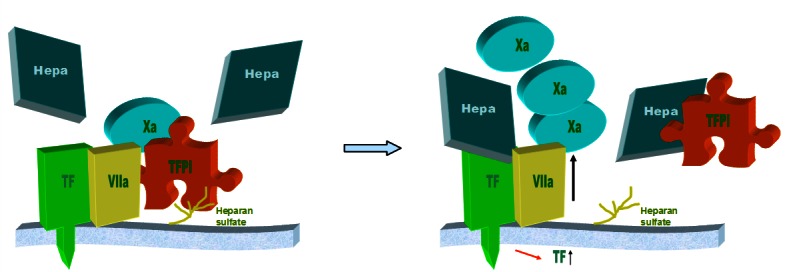
**A model of the interaction between heparanase (Hepa), TF, and TFPI.** Heparanase interacts with TF resulting in increased generation of factor Xa and enhancement of the coagulation system. Heparanase also up-regulates TF expression and releases TFPI from the cell surface, rendering the cell surface highly procoagulant. TFPI and heparanase may circulate as a complex in the plasma.

Platelets and tumor cells have abundant amounts of heparanase.^53^ Activation of the coagulation system, including platelet activation, occurs in malignant and angiogenic processes.^69^ Heparanase is directly involved in activation of the coagulation system by enhancing factor Xa production in the presence of the TF/VIIa complex. Additionally, heparanase released from activated platelets and tumor cells induce up-regulation of TF in the cells. Heparanase-mediated release of TFPI from the cell surface, together with its induction of TF, renders the cell surface highly procoagulant. Heparanase may also form complexes with TFPI and circulate in the plasma, possibly binding to endothelial cells and other intravascular components, i.e. platelets and microparticles. These aspects are depicted in Figure 1.

**Figure 2:**
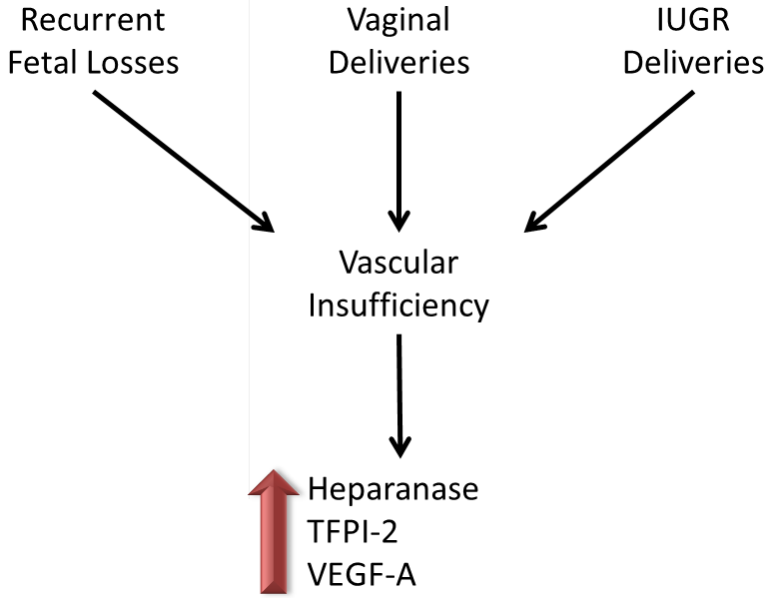
**Heparanase, TFPI-2, and VEGF-A are elevated in placentas with vascular insufficiency.** A schematic summary of two studies^73^,^74^ implying elevated levels of heparanase, TFPI-2, and VEGF-A in placentas of women with recurrent fetal losses, IUGR deliveries, and normal vaginal deliveries. In these three conditions vascular insufficiency occurs. As heparanase has a procoagulant role, it potentially can contribute to thrombosis in these placentas.

Pregnancy causes an acquired hypercoagulable state, and women with a prior tendency to thrombosis may present with clinical symptoms of placental vascular complications. Maternal thrombophilia can be associated with placental vascular events, although 30%–50% of vascular gestational pathologies cannot be accounted for by the currently available tests for thrombophilia.^70^ Thus, an understanding of the hemostasis in the placenta, especially the dominant factors that regulate the delicate hemostatic balance throughout pregnancy, is essential. Heparanase is abundant in the placenta and was originally cloned from placenta tissue. Additionally, estrogen was found to up-regulate heparanase gene expression in human endometrium^71^ and breast cancer.^72^ Recently, we investigated the role of heparanase in the placenta, focusing on its effect on TF, TFPI, TFPI-2, and VEGF-A.^73^,^74^ In these two studies placenta samples of women with recurrent abortions and thrombophilia (weeks 6–10) were compared to control cases of pregnancy terminations and placentas of normal vaginal deliveries, and intrauterine growth-restricted (IUGR) babies were compared to control cases of elective cesarean sections, applying real-time RT-PCR and immunostaining. Sections obtained from miscarriages and vaginal and IUGR deliveries revealed increased (2–3-fold) levels of heparanase, VEGF-A, and TFPI-2 compared to placentas from controls in maternal as well as in fetal placenta elements. A possible common denominator of the cases is vascular insufficiency: in vaginal deliveries lasting intermittently for a few hours; in miscarriages and IUGR babies it may represent a prolonged state. As heparanase directly activates the coagulation system,^56^ increased heparanase found in the placentas may contribute to placental vascular complications as summarized in Figure 2.

## CONCLUSIONS

Heparanase was recently revealed as an important modulator of blood coagulation. The elevation of heparanase levels in human tumors, together with the prothrombotic state of most neoplasms, suggests possible clinical relevance of the procoagulant function of heparanase. In addition its increased levels in pregnancy vascular complications accentuate heparanase significance in other proangiogenic states. In order to augment the understanding of heparanase we lately developed an assay to evaluate heparanase procoagulant activity in the plasma,^75^ enabling further extensive research in the field. Targeting domains of heparanase that mediate its enzymatic activity-dependent and independent functions may prove beneficial for patients with proangiogenic and prothrombotic conditions.

## Abbreviations:

ALL: acute lymphoblastic leukemia;
AML: acute myeloid leukemia;
ECM: extracellular matrix;
HS: heparan sulfate;
HUVEC: human umbilical vein endothelial cell;
IUGR: intrauterine growth-restricted;
LMWH: low-molecular-weight heparin;
MM: multiple myeloma;
SNPs: single nucleotide polymorphism;
TF: tissue factor;
TFPI: tissue factor pathway inhibitor;
VEGF: vascular endothelial growth factor.
